# Artificial intelligence in diagnostic pathology

**DOI:** 10.1186/s13000-023-01375-z

**Published:** 2023-10-03

**Authors:** Saba Shafi, Anil V. Parwani

**Affiliations:** Department of Pathology, The Ohio State University Wexner Medical Center, E409 Doan Hall, 410 West 10th Ave, Columbus, OH 43210 USA

**Keywords:** Artificial intelligence, Pathology, Future, Algorithms

## Abstract

Digital pathology (DP) is being increasingly employed in cancer diagnostics, providing additional tools for faster, higher-quality, accurate diagnosis. The practice of diagnostic pathology has gone through a staggering transformation wherein new tools such as digital imaging, advanced artificial intelligence (AI) algorithms, and computer-aided diagnostic techniques are being used for assisting, augmenting and empowering the computational histopathology and AI-enabled diagnostics. This is paving the way for advancement in precision medicine in cancer. Automated whole slide imaging (WSI) scanners are now rendering diagnostic quality, high-resolution images of entire glass slides and combining these images with innovative digital pathology tools is making it possible to integrate imaging into all aspects of pathology reporting including anatomical, clinical, and molecular pathology. The recent approvals of WSI scanners for primary diagnosis by the FDA as well as the approval of prostate AI algorithm has paved the way for starting to incorporate this exciting technology for use in primary diagnosis. AI tools can provide a unique platform for innovations and advances in anatomical and clinical pathology workflows. In this review, we describe the milestones and landmark trials in the use of AI in clinical pathology with emphasis on future directions.

## Background

### Milestones and landmark trials in computational pathology (Figure 1 and Figure 2)

Some important milestones in computational pathology are as follows:

1950: Alan Turing conceived the idea of using computers to mimic intelligent behavior and critical thinking [1].

1956: John McCarthy coined the term artificial intelligence (AI) [2, 3].

1959: Arthur Samuel coined the term machine learning (ML) as “the ability to learn without being explicitly programmed” [4].

1960: Prewitt and Mendelsohn scanned images from blood smear and reported a method to convert optical data into optical density values [5–7].

1965: Computerized image analysis of microscopy images of cells and chromosomes by Judith Prewitt and Mortimer Mendelsohn [8].

1986: Term deep learning (DL) coined by Rina Dechter [9].

1988: Convolutional neural network (CNN) invented by Yann LeCun [10].

1990: Whole slide scanners introduced [11, 12].

1998: Tripath becomes the first company with an automated PAP smear screening product to receive FDA approval [13–15].

2003: Cytyc received FDA approval for their ThinPrep Imaging System [13–15].

2013: Development of photoacoustic microscopy imaging technique [16].

2014: Ian Goodfellow introduced generative adversarial network [17].

2016: MUSE microscopy technique invented to enable high resolution imaging without tissue consumption [18].

2017: Philips receives approval for a digital pathology whole-slide scanning solution (IntelliSite) [19].

2018: FDA permits first medical device using AI to detect diabetic retinopathy in adults (IDx DR) [20].

2021: FDA authorizes the first AI-based software to detect prostate cancer (Paige Prostate) [21].

### Role of AI in pathology: a brief overview

**Fig. 1:**
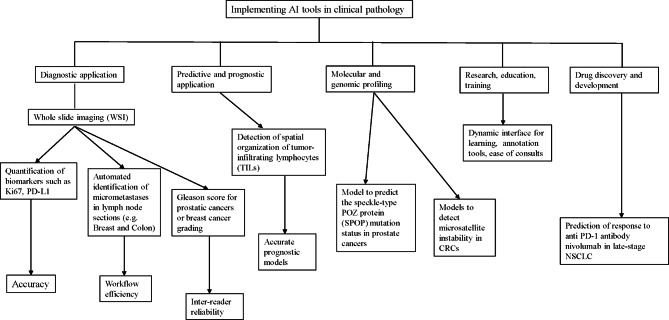
Milestones in digital and computational pathology with depiction of three “revolutions” in the field

**Fig. 2:**
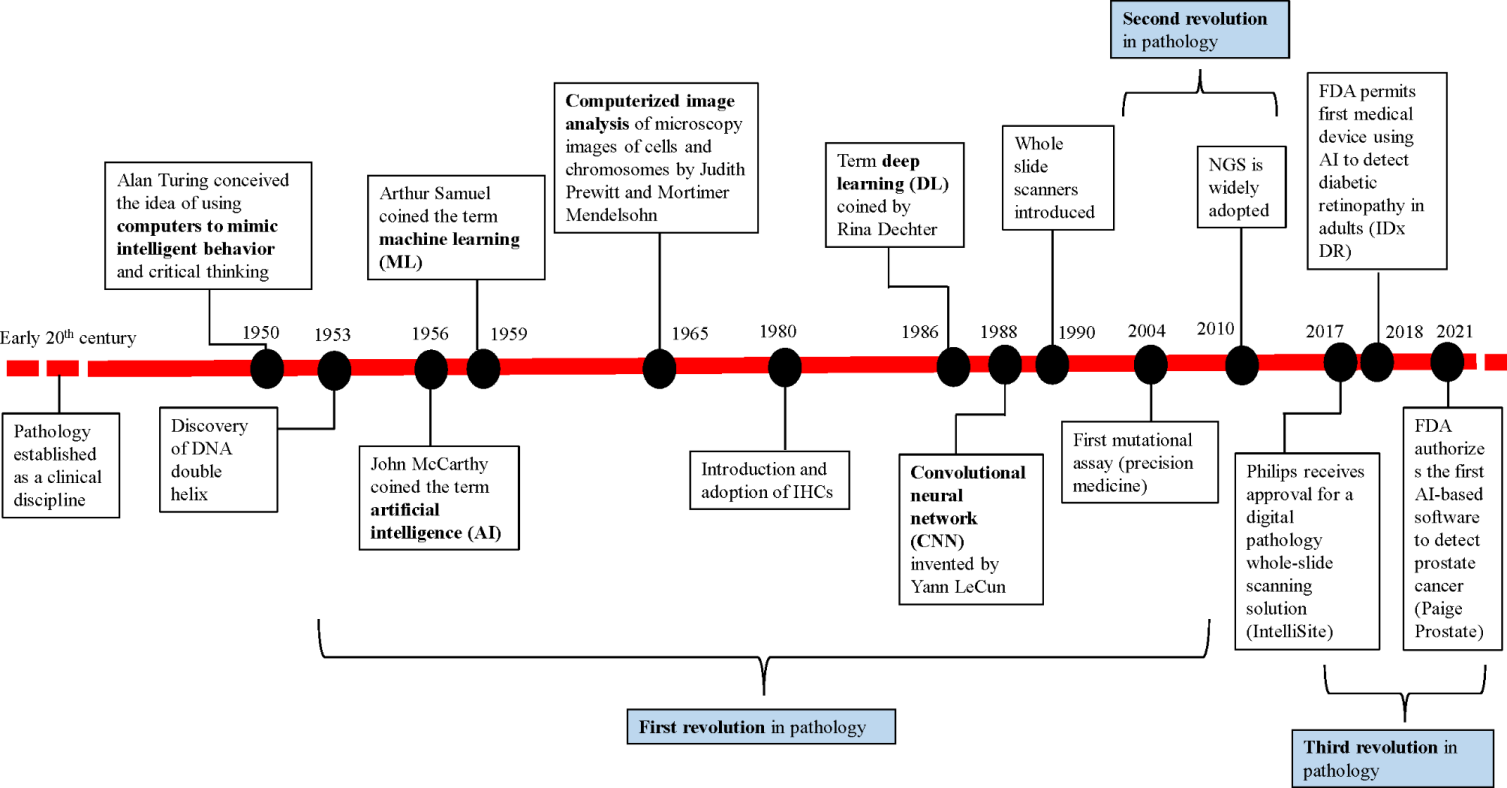
Schematic representation of the how artificial intelligence (AI) can be applied in the practice of pathology

Machine learning (ML)-based approaches are based on the machine “learning” to make predictions based on the input data and algorithms and falls within the broad ambit of AI [22, 23]. Deep learning (DL) network consists of an input layer, multiple hidden layers, and an output layer, recapitulating the human neural architecture. The hidden layers can recreate newer visualizations of the image and with appropriate number of repeats which can identify representations allow for the differentiation between interesting features [24, 25]. AI methods are increasingly being applied in pathology practice for a wide variety of image analysis and segmentation type of tasks [26, 27]. These include trivial tasks, such as object recognition of cells etc., as well as more complex actions such as using image pattern recognitions for predicting disease diagnosis, prognosis, and therapeutics [28–50]. The main underlying principle of these AI approaches is to extract image patches which can be used to providing training to algorithms [28–35]. AI has helped with creating morphometric analysis methods which can facilitate quantitative histomorphometry (QH) analysis approaches for detailed spatial interrogation (e.g., capturing nuclear orientation, texture, shape, architecture) of the entire tumor histologic landscape from a standard hematoxylin and eosin (H&E) slide [51]. These AI applications primarily aim to automate tasks that are time-consuming for the pathologist, thereby aiding fast and reliable diagnoses by utilizing the time saved on high-level decision-making tasks [28, 29, 33–35, 52–56]. Thus, AI technology can be used to support the overall reporting system, speed up reporting time and measure morpho-biological features more objectively. AI-aided reporting of certain features or lesions will also enable pathologists to focus on challenging cases and meet the increasing workload demands. Implementation of such technology in the workflow of pathology service is not to replace the human resources including pathologists, and laboratory technicians, but to provide support for them, assist them and augment diagnostic and performance efficiency with better allocation of resources, increased cost-effectiveness of the service and more consistent pathology reviews (Figs. 1 and 2) [57].

## Main text

### Hand-crafted feature-based approaches

ML algorithms can be developed either using intrinsic domain knowledge of pathologists and oncologists (domain-inspired features) or without this inherent domain knowledge (domain-agnostic features). This process is called feature engineering [33, 58, 59]. An example of domain inspired feature is the co-occurring gland angularity feature presented by Lee et al. which involved computing the entropy of gland directions within local neighborhoods on tissue sections. Aggressive risk prostate cancer had more chaotically arranged glands compared to low to intermediate risk cancer. Consequently, the entropy associated with these “gland angularity features” (GAF) was found to be higher in aggressive and lower in indolent disease [39].

Image characterization across several disease and tissue types can be better depicted using domain-agnostic features. Examples include nuclear and gland shape and size, tissue texture and architecture. Automated Gleason grading of prostate pathology images has been arrived at using a series of wavelet and tissue texture features enabling machine-based separation of low and high Gleason grade prostate pathology images. Hence both domain-agnostic and domain-specific hand-crafted feature-based approaches have been used for the diagnosis, prognosis, grading, and prediction of response to therapy for various cancers such as breast, prostate and brain tumors [60].

**Fig. 3:**
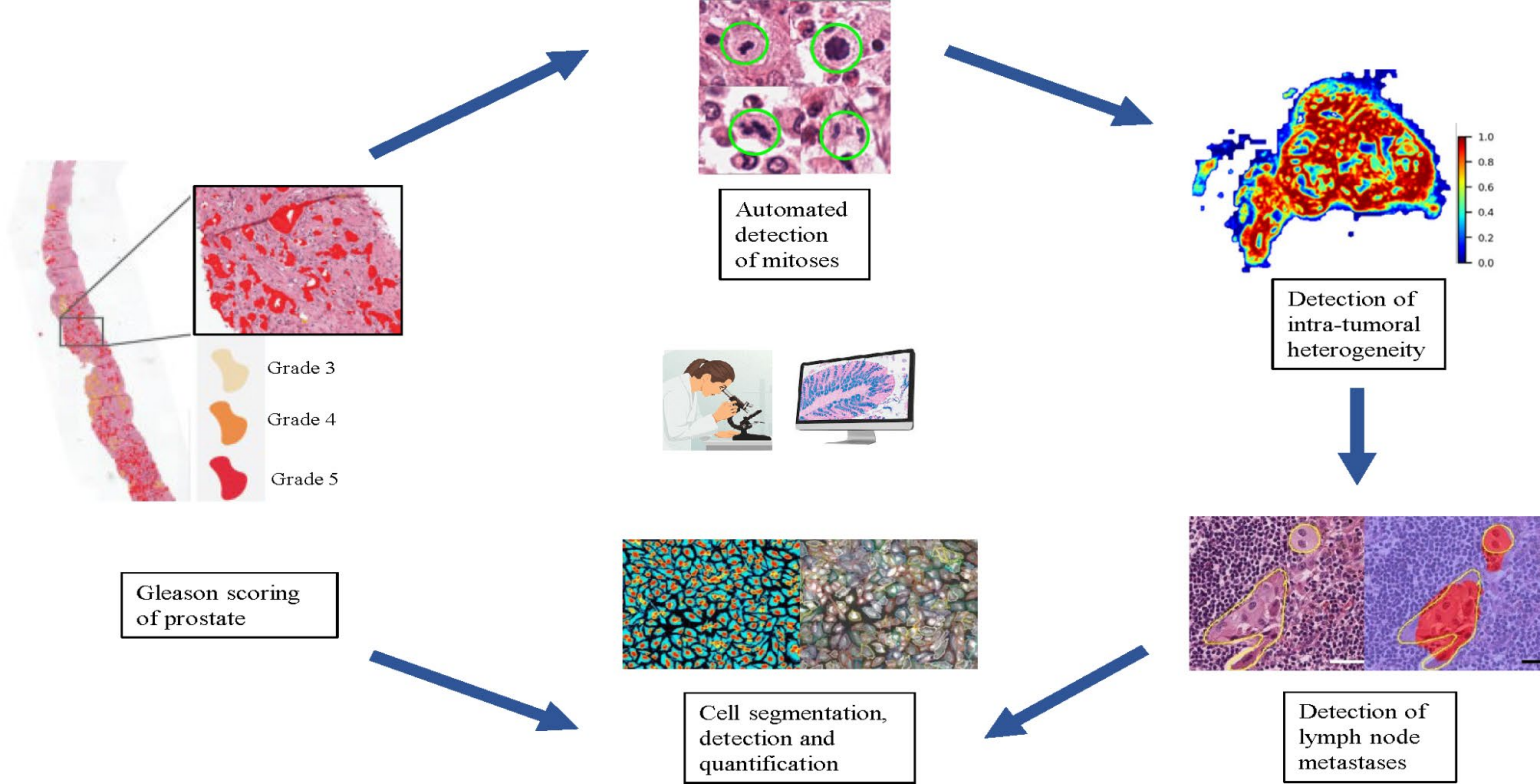
Depiction of artificial intelligence (AI) and machine learning approaches currently used by pathologists to analyze images from tumors

Handcrafted and unsupervised features have several advantages and limitations. Handcrafted features are more transparent and intuitive to the pathologist or oncologist. Domain-inspired features require a strong foundational knowledge of the pathological process and its manifestation within the tissue and thus are more challenging to develop. The unsupervised feature generation-based approach of deep learning strategies lacks feature interpretability though it can be applied quickly and seamlessly to any domain or problem [33, 40, 41, 51, 58, 59, 61, 62] (Fig. 3).

### Implementation of AI tools in clinical pathology practice

#### Applications of AI in diagnostics

Recently, promising strides in AI have opened new vistas for significantly altering the way cancer is diagnosed and classified [63]. Several advances have been made in incorporating AI tools to the diagnostic workflow in pathology practice. AI approaches have been used in a variety of tasks such as object recognition, detection, and segmentation [28–38]. WSIs can be used to extract several features using computer vision algorithms, thereby enabling diagnostic predictions [64–69]. Several AI tools are increasingly being used to provide information that is gleaned difficult for the pathologist to identify [66, 68, 69]. Examples include accurate objective assessment of immunohistochemical biomarkers such as Ki67, PD-L1, quantification of cells, evaluation of spatial arrangement of cells, expression, density, and pattern of distribution [32, 70]. AI can also be used to detect isolated tumor cells in lymph nodes suspicious for metastatic carcinoma, increasing sensitivity of detection in a time-efficient manner. Additionally, AI tools can help standardize scoring criteria in several tumors, such as Gleason score for prostatic cancers or breast cancer grading, where the morphological features are represented on a spectrum of a continuous biological process [26, 71, 72]. Another striking application of AI search tools is the content-based image retrieval (CBIR) which enables pathologists to search for images similar to the image-in-question from a repository of large histopathology database. This is especially important in guiding pathologists to diagnose rare and complex cases which they might occasionally come across in their clinical practice The images retrieved from the database reflect similarities in associated histopathological features rather than mere image similarity. Hence, CBIR makes it easier to render a correct diagnosis in a timely fashion for seemingly difficult cases [73, 74].

Diagnostic algorithms can be incorporated into digital pathology workflow as independent reporting algorithms, as diagnosis-aided tools, and as automated quantifiers of specific features. Independent reporting algorithms can provide diagnosis and automated reports without any input from the pathologists. Screening algorithms can identify normal tissue like colonic, gastric, breast etc. from biopsies. However, it is important to consider the wide varieties of normal tissue during the algorithm development pathway, to avoid missing rare microlesions (such as benign mimickers of cancer) or focal lesions which are rare variants of cancer. Diagnosis-aided tools include algorithms that assess one of the various histological features such as tumor grade, type, and extent. Accurate pathological diagnoses involve assessment and combination of multiple features by the trained human eye. The utility of these AI algorithms is determined by its ease of incorporation into the diagnostic workflow and the added value it brings to the pathologists’ diagnoses. This can be assessed based on the features assessed and the time required to provide results as well as on its accuracy. As an example, breast cancer grading algorithms have the potential advantage of objectivity, inter-reader reliability and prognostic clarity compared to the inter-observer variability seen in clinical practice. Hence in this case, the added value of such an AI algorithm would be better reproducibility rather than efficiency [56, 72]. Therefore, it is essential to not merely use AI algorithms, but to use them intelligently [27]. For instance, for the detection of lymph node metastases, AI can have superior performance when serving as a pathologist assistive tool, underscoring the importance of the context of intended clinical use. Automated quantification of immunohistochemical markers has generated considerable interest as increasing efforts are being made to not only provide an objective estimation of these markers but also give an estimate of their predictive and prognostic value. While the manual estimation of breast cancer receptors might take only a few minutes by an experienced pathologist, it can be made more efficient and reproducible by an automated method [72].

Many digital image analysis (DIA) platforms have been adopted to aid pathologist assessments especially for quantitative biomarker evaluations [75]. Amongst the first open-source tools for image analysis was ImageJ, developed in 1997 by the National Institute of Health (Bethesda, Maryland, USA). In 2006, CellProfiler software was published which provided supervised machine learning-based classification to perform imaging-based diagnoses. Another accessible platform being increasingly used for image analyses is QuPath, first published in 2017. The software functions to provide unsupervised machine learning-based cell detection and supervised classification of whole slide images, tumor identification and quantitative biomarker assessment. Ventana Companion Algorithm image analysis software has received approval from CE and US IVD for Roche IHC assays in breast cancers for the assessment of breast biomarkers (ER, PR, HER2, Ki67 and p53). The Tissue Phenomics software was created by AstraZenecain 2014 and applied to clinical programs in immune oncology for identifying biomarkers. HALO (Indica laboratories) has developed modules for quantitative immunofluorescence analysis primarily for research purposes. Scoring of Ki67, ER, PR, CD3/4/8/15/20 and TILs has been made possible using Cognition Master Professional Suite platform developed by VMscope. QuantCenter, a framework for 3DHISTECH image analysis applications, provides modules for tissue classification, IHC quantification and molecular pathology. The rapid emergence of digital image analysis solutions and integrated platforms has resulted in the need for validation and standardization of these tools before they can be approved in the diagnostic setting [76–78].

A milestone study in computational pathology is The CAMYLEON16 challenge, the first major challenge on computer-aided diagnosis in histopathology using whole slide images. H&E images from sentinel lymph nodes of breast cancer patients were used with the aim of identifying metastasis. With no time constraint, DL algorithms showed comparable performance to a pathologist in detection of micrometastasis. To simulate a clinical practice setting, time constraint was imposed, which demonstrated an outperformance by algorithm over manual evaluation by 11 pathologists [28]. Differentiation between benign and malignant tumors using a supervised ML model trained on whole slide images obtained by fine-needle biopsy has been made possible [79]. Veta et al. highlighted the prognostic value of features such as nuclear shape or texture in male breast tumors using tissue microarray (TMA) [80]. In their study, Lee et al. used WSIs from prostate cancers to describe gland angularity feature (GAF) which was related to the degree of disarray of the glandular architecture. GAF demonstrated high association with advanced stage prostate cancers. Nuclear pleomorphism, orientation and architecture have been employed to develop hand-crafted features in the tumor and benign tumor-microenvironment. These features used in tandem with a ML model was designed to predict the chance of recurrence within the 5 years of post-operative period [39]. Similarly, variations in nuclear shape and texture were used in oral cavity squamous cell carcinomas to stratify patients into risk categories predictive of disease-free survival (DFS). It was further elucidated that patients with estrogen receptor (ER)-positive breast cancer with short-term survival (< 10 years) could be distinguished from those with long-term survival (> 10 years) based on a combination of nuclear shape and orientation features [62].

Another key milestone in the field of DP/AI has been the PANDA challenge, the largest histopathology competition thus far. Nearly 1,290 developers joined hands and used 10,616 digitized prostate biopsies for the development of AI algorithms for Gleason grading. The algorithms submitted were selected based on the level of accuracy achieved compared to pathologist on independent cross-continental cohorts. In United States and European external validation sets, the algorithms achieved agreements of 0.862 (quadratically weighted κ, 95% confidence interval (CI), 0.840–0.884) and 0.868 (95% CI, 0.835–0.900) with expert uropathologists. However, in order for these algorithms to be applied clinically, across different patient populations, laboratories, and reference standards, prospective clinical trials evaluating AI-based Gleason grading is warranted [81].

To date, the most widely used DL algorithms in pathology applications is convolutional neural networks (CNNs). Defined as a type of deep, feedforward network, CNN consists of multiple sequential layers (convolutional sheets) that can calculate an output from an input (such as an image), by hierarchically deconstructing the image into low-level cues. Aggregation of these low-level cues, such as edges, curves, or shapes results in the construction of a high-order structure to identify features of interest [29, 30, 82–87]. Araujo et al. used CNN for the classification of WSI of breast cancers into benign, malignant, in-situ or invasive. CNN was also shown to have performance comparable to dermatopathologists in distinguishing benign lesions such as seborrheic keratosis from keratinocyte carcinoma and benign nevi from malignant melanoma [83]. Tschandl et al. demonstrated that CNN had similar diagnostic accuracy as humans in correctly classifying pigmented skin lesions using digital dermatoscopic images. These findings, among others, have established the role of AI based methods in diagnostic practice [88].

#### Predictive and prognostic applications of AI

AI can be used to predict prognosis and therapeutic responses based on histological features [89, 90]. Directly linking images with several features of tumor, surrounding microenvironment and genetic profiles with survival outcomes and treatment response for adjuvant/neoadjuvant therapy could provide important information in a concise manner. Integrating myriad morphological features, such as tumor histological patterns and tumor microenvironment patterns into a single prognostic index can be difficult for humans [26, 91]. However, image-based AI tools can provide a novel classification system depicting clinical outcome, probability of recurrence or metastases and therapeutic response by correlating important histological features such as tumor morphology, stromal architecture, nuclear texture, and lympho-vascular invasion etc. Prediction of clinical outcome using graphical approaches for evaluation of architectural organization and spatial configuration of different types of tissues has resulted in considerable interest [63]. Wang et al. trained a ML model using nuclear orientation, nuclear shape, texture, and tumor architecture to predict recurrence in early-stage non-small cell lung cancer (NSCLC) from HE stained TMA slides. Their model’s prediction was shown to be an independent prognostic factor resulting in 82% and 75% accuracy for prediction of recurrence in two validation cohorts [61] (Fig. 3).

The prognostic implications of AI based tools were also highlighted in 2018 by Saltz et al. who used a convolutional neural network (CNN) to augment pathologist feedback for the automatic detection of spatial organization of tumor-infiltrating lymphocytes (TILs) in images from The Cancer Genome Atlas. Their findings revealed this feature to be prognostic of outcome in 13 cancer subtypes [92]. A similar study conducted by Yuan et al. described a model to analyze the spatial distribution of lymphocytes with respect to tumor cells on triple-negative breast cancer WSIs. Not only did they identify three different categories of lymphocytes, so did they find a direct correlation between late recurrence and the spatial distribution of immune cells in ER-positive breast cancers [93]. CNN has also applied to breast cancer TMAs for histological and molecular characterization. Automated detection of mitotic figures in breast cancer WSIs using CNN has revealed a significant difference between a high versus a low Oncotype DX-defined risk of disease recurrence [72]. Similar prognostication study in colorectal cancers using CNN-based approaches was performed by Geessink et al. Employing pathologist-defined ‘stromal hotspots,’ CNN enabled quantification of ‘tumor-to-stroma’ was seen to be independently prognostic for disease free survival [94].

A seminal multi-institutional study published by Beck et al. used a staggering number of morphological and spatial features (6642) to train a prognostic model in breast cancers and demonstrated these features to be associated with overall survival (OS) [95]. A similar study in human papillomavirus-positive oropharyngeal cancers showed that combining nuclear features of the stromal and the epithelial compartments enabled prediction of the likelihood of progression of these cancers [96]. A related study in prostate cancers indicated the need for population-specific features while designing models, as significant differences in nuclear features of the stromal compartment were noted between Caucasians and African cohorts. The importance of such population-specific models was also highlighted by greater accuracy in calculating recurrence in a validation cohort of African American population by using an algorithm trained in a cohort of similar racial demographics compared to that trained in a mixed population [97].

#### AI as predictor of molecular and genomic profile

Recent advancements entail using H&E images to predict genetic alterations by deep learning algorithms. AI tools can be used to derive information about tumor genetics/genomic profiles from morphology and thus help in understanding underlying cancer biology [98]. Molecular-based testing for prognostic purposes that incorporates information from multiple parameters are already available, e.g., the mRNA-based oncotype test [91]. Schaumberg et al. devised a model to predict the speckle-type POZ protein (SPOP) mutation status in prostate cancers which showed an area under curve (AUC of 0.74 and AUC of 0.86 in two independent cohorts. Similar attempts have been made to predict commonly mutated genes in other tumor types [99]. Coudray et al. were able to generate a CNN model that could predict mutations in KRAS, EGFR, TP53, FAT1, STK11 and SETBP1, with high accuracy (AUC between 0.733 and 0.856). Similar approaches have been used for obtaining information regarding microsatellite instability from H&E images in colorectal and gastric cancers [100]. One such study by Kather and colleagues used a deep learning approach for elucidating microsatellite instability in a total of 1616 H&E images of both frozen and formalin fixed specimens with high accuracy (AUCs between 0.69 and 0.84 in five cohorts) [101].

While the identification of association between morphological patterns and tumor genetics seems to be straightforward, integrating mega volumes of genomic data such as next-generation sequencing (NGS) can be challenging. Studies highlighting the impact of combining NGS data with other features are warranted before implementing such algorithms in clinical diagnostic practice. What further complicates the picture is the lack of in-depth knowledge about the interaction between imaging and genomic features. Although integrating the imaging and molecular features can provide a comprehensive view of individual tumors, however, development, training, and validation of models capable of tackling such sophisticated multidimensional data remains a challenge [57].

#### Utility of AI in research, training, and education

AI tools provides critical tools to enhance pathologists’ training, provide helpful annotations and other interactive functions to create a dynamic teaching environment for trainees. This can help integrate a strong knowledge of morphology with the use of novel approaches and advanced technologies in enabling the practice of high-quality personalized and precision medicine. Whole-slide imaging is already used for teaching at conferences, virtual workshops, presentations, and tumor boards [26, 102]. The Ohio State University Wexner Medical Center has incorporated the use of a “digital cockpit” for fully digital sign-outs. Residents regularly preview digital slides using the Philips integrated management system (IMS). The annotation tools enable viewing, panning, and zooming enhanced digital slides, encircling regions of interest, including a single cell under question, thereby creating a more interactive learning interface. The clinical and research registries, the organ-based databases, our exceptional laboratory information system (LIS, colloquially called “beaker”) as well as the synoptic reporting templates are excellent examples of bioinformatics driven tools used in the everyday practice of pathology. The university also leverages several of its add-on components to enable integration of WSI into the LIS. This underscores the state-of-the-art incorporation of digital pathology tools in clinical workflow [103, 104]. The use of Visopharm AI tools enables swift detection of isolated tumor cell metastases in lymph nodes in difficult cases. Integrating such AI tools in the daily sign-out workflow can supplement key information for the trainees to come up with a list of differential diagnosis and potential auxiliary tests that can be subsequently ordered, thereby honing their diagnostic skills. It also provides relevant educational resources which can potentially improve resident training. Such educational models can be complementary to the conventional educational processes provided by the pathologists and can be adopted by other institutions. Not only has it improved the in-house training and inter-subspecialty consults, so has it made it a lot easier to collaborate with other institutions and provide efficient consults and second opinions on challenging cases [26, 103, 104].

#### Role of AI in drug discovery and development

Immune checkpoint inhibitors (ICIs) have led to a paradigm shift in treatment of various cancers over the past few years. However, many patients receiving ICIs do not respond to this therapy, and this has resulted in the potential need for combining AI with digital pathology to stratify patients based on likely therapeutic benefit [105]. A study on recurrence risk stratification of early-stage non-small cell lung cancers (NSCLC) based on nuclear and perinuclear features (shape, orientation, and spatial arrangement) was conducted by Wang and colleagues. High-risk patients were potential candidates for adjuvant chemotherapy. AI tools, such as hand-crafted ML approaches, can also be used to predict therapeutic response to targeted agents, ICIs, and chemotherapeutic drugs. One such study by Wang et al. described the prediction of response to anti programmed cell death 1 (PD-1) antibody nivolumab in late-stage NSCLC using spatial orientation of nuclei and TILs [44, 61, 106].

### Roadblocks and challenges preventing AI application

#### Ethical principles and AI

In 2016, Wilkinson et al. highlighted the need to improve the infrastructure supporting the reuse of scholarly data and came up with the FAIR guiding principle for scientific data management and stewardship. Data should be easily accessible, operator-independent, and reusable to ensure stringent data management. This is essential for knowledge discovery and innovation, and ensures reusability of data by the community after publication (Table 1) [107].

**Table 1:** An overview of the challenges and roadblocks encountered during various steps of using artificial intelligence (AI) tools in pathology workflow

Process involved in integration of AI tools in pathology	Challenges and roadblocks
Identification of needs	• Incorrection assessment of end-user and demands • Small market size of AI usage • Lack of awareness of possibilities of use
Collaborative inter-disciplinary efforts	• Lack of coordination between different players • Discordance in goals of participants
Study concept, design	• Scientific background/rationale • Funding • Ethical approval
Development of algorithmic models	• Pre-analytical and analytical factors • Lack of objective ground truth
Optimization, validation, and standardization	• Lack of appropriate validation dataset • Overfitting
Interpretability	• Lack of interpretability and generalizability • Black-box issue
Data curation	• Difficulty in obtaining well-curated, annotated data
Regulation/approval	• Lack of clear-cut regulatory guidelines
Installation	• Pathologists’ resistance to changes in old workflow • IT infrastructure investment and overhead costs
Accreditation	• No external quality assurance scheme • Unestablished audit cycles
Reimbursement	• Lack of dedicated procedure codes
Clinical adoption	• Lack of FDA approval for use of AI • Skepticism among pathologists and oncologists
Computation system and data storage	• Need for powerful, high specification hardware • Cost-benefit ratio considerations

#### Validation of algorithms and overfitting

AI algorithms need rigorous multi-institutional validation before they can be clinical implementation. This usually requires application of the algorithmic approach/model on a training/learning discovery set, followed by confirmation of results on validation set. Current AI algorithms are mainly established on small-scale data and images from single center, augmented by random rotation and flipping, color jittering, and Gaussian blur. The training set should be well balanced in terms of equal representation from all categories of interest. Once the algorithm is trained after several iterations on the discovery dataset, further optimization is performed on the validation dataset. This process can be quite laborious and challenging and acquisition of pertinent datasets/cohorts might be cumbersome [91]. In a study performed by Zech et al. it was shown that a CNN algorithm for detection of pneumonia performed significantly poorer when it was trained using data from one institution and validated independently using data from two other institutions than when it was trained using data from all three institutions, thereby highlighting the need for robust validation of AI algorithms using multi-institutional data before clinical adoption [108]. ‘Overfitting” is when AI algorithms, trained on one dataset, have limited applicability to other datasets [109]. It can be difficult to find well-curated, accurate WSI reference datasets across cancer subtypes with annotated cancerous regions for algorithmic standardization. Furthermore, differences in pre-analytical and analytical factors, such as slide preparation techniques, scanner models and digitization processes, between different centers must be considered while using applying these AI tools. To ensure the generalizability and robustness of the AI algorithms, stringent quality assurance and standardization needs to be done at regular intervals. This requires development of large databases and repositories of annotated WSIs validated, corrected, and updated by a team of expert pathologists (Table 1) [91, 110].

#### Interpretability and the ‘black box’ problem

The ‘black box’ problem is the inability of deep learning algorithms to demonstrate how they arrive at their conclusions [111, 112]. Despite obvious advantages of accuracy and efficiency, deep neural networks face sharp criticism due to lack of interpretability, which forms a huge roadblock in clinical adoption. Several studies aimed to overcome this skepticism used post hoc methods to comprehensively analyze the outputs of AI algorithms. However, post hoc analyses of deep learning methods seem superfluous as additional models should not be required to explain how an AI model works. Due to their development in conjunction with domain experts, hand-crafted AI approaches offer an advantage of better interpretability. To increase interpretability, researchers have integrated DL algorithms and hand-crafted ML approaches to come up with ‘fusion’ approaches. Be as it may, engineering both these methods is challenging and time-consuming and requires both oncologists’ and pathologists’ inputs. One such ‘fusion’ method to predict disease recurrence was described by Wang et al. who used a DL approach for nuclear segmentation in H&E images of NSCLC followed by application of hand-crafted method based on nuclear shape and texture. Future strategies are warranted to increase the interpretability of AI algorithms before they can confidently be used in the clinical setting [51, 111, 113].

#### Quality of data

For optimal performance of AI-based approaches, it is highly important that the input data be of optimal quality and quantity. The highest predictive accuracy is reached when the training data has optimal signal-to-noise ratio, is well-curated and comprehensive. The importance of high-quality data is highlighted in the work of Doyle et al. that used an AI tool for automatic detection of prostate cancer in WSIs [110]. An increase in magnification resulted in a decrease in the overall performance of the model due to loss of granularity at increased resolution. Majority of existing slide scanners have a maximum capability to scan at ×40. While higher resolution images (>×20) can be scaled down to be used by an algorithm trained at a resolution of ×20, considerable loss of data fidelity can occur with the use of an AI approach developed at ×40 when the maximum scanning resolution available is ×20. Hence, ensuring data fidelity is of paramount importance in order to standardize the evaluation of the performance of AI algorithms [52, 110, 114].

#### Computational system, data storage and cost-benefit ratio

It is essential to have a powerful high specification hardware for processing and analyzing images as well as ample scalable data storage for storing these large size files (about 1000 times the size of an X-ray). Buying cloud storage platforms might be costly as well as have challenges in in the massive bandwidth required to transmit gigapixel-sized WSI images into data clouds. Additionally, cloud storage requires uninterrupted fast wi-fi communication between end-users and the cloud. Universal adoption of 5G would improve speed and address some of these difficulties in the future [115]. The cost of procurement, implementation, and operational costs of AI may be a limiting factor, especially for small laboratories. The high initial cost of the scanners and additional hidden costs of training of staff and pathologists, technical support, digital slide storage systems, and regulatory or licensing costs incurred may be prohibitive to the adoption of AI in clinical practice [116]. Another cost consideration is the robust IT support for telepathology. A study found the cost-benefit analysis at a large-volume academic center with slides in excess of 1.5 million to be projected $1.3 million savings over a 5-year period. Before any meaningful inferences can be drawn, cost-benefit studies need to be performed in low-resource settings and small pathology laboratories [117]. In stark contradistinction to radiology, where digital systems obviate the need of making films, WSI in pathology does not reduce the laboratory’s workload since glass slides still need to be prepared to be scanned, thereby raising concerns about the justification for this additional step [118].

#### Technological issues

Scanning the entire slide is a laborious and time-consuming process with variable scanning times ranging between 1 and 5 min for a small biopsy, 5–20 min for a surgical specimen and 3–5 min for a liquid-based cytology smear. Additionally, most of the current scanners require massive data storage capacity with 1-mm^2^ at × 40 magnification resulting in a file size of 48 megabytes! To overcome this, most WSI platforms resort to image compression algorithms (JPEG, JPEG 2000, LZW) to reduce size significantly. The disadvantage of this compression is the introduction of image artefacts which can compromise overall pixel quality (Table 1) [118].

#### Regulation, reimbursement, and clinical adoption

Before an AI algorithm can be used in the clinical setting, it is essential to get clearance by the regulatory bodies. For getting approved, a clear description of how the software works must be provided, especially for DL-based algorithmic approaches that are perceived as a ‘black-box’ lacking interpretability. Depending upon the country, The Food and Drug Administration (FDA), the European Medicines Agency (EMA) and other regulatory agencies lay down stringent guidelines and frameworks for ensuring scientific rigor of the reported metric. The FDA approval of medical devices is based on a three-class system with Class I devices supposed to have the lowest risk and Class III devices deemed to have the highest risk. AI-based models fall within class II or III, with class III requiring a rigorous premarket approval. 510(k)-approval pathway and De Novo pathway are some other ways of getting AI algorithms approved. Be as it may, the process is highly rigorous and comprehensive [119–121]. In 2017, Phillips got De Novo approval for introducing the IntelliSite Pathology Solution [19]. This was followed by PAIGE.AI’s FDA approval as Breakthrough Device in 2019 [122]. Interestingly, no FDA approval was sought for OncotypeDX for breast cancers since it was a Clinical Laboratory Improvement Amendments (CLIA)-certified central test assay. Laboratory Developed Tests (LDTs) are usually complex and due to “black box” issue, the College of American Pathologists (CAP) has requested for a more stringent FDA regulation for such high-risk prognostic and predictive tests. At present, no dedicated procedure codes exist for the use of AI approaches in digital pathology with diagnostic or prognostic intent. Once AI tools receive FDA approval, new procedure codes will need to be established to bill patients [91, 123]. CAP, working with the American Medical Association (AMA) CPT Editorial Panel, has successfully advocated for the inclusion of 13 new digital pathology add-on codes to be effective January 1, 2023. The new codes have been accepted for Cat III - Digital Pathology: 0751T to 0763T. These new digital pathology CPT codes will be used to report additional clinical staff work and service requirements associated with digitizing glass microscope slides for primary diagnosis (Table 1) [124].

#### Pathologists’ dilemma-to use or not to use

A key roadblock for the incorporation of AI in clinical practice comes from an apprehension about the change in workflow. This is partly because of lack of interpretability and partly due to the somewhat unclear question of performance thresholds of AI algorithms. While there is evidence of decreased error rates and improved performance using a combination of DL-based model predictions with pathologist diagnoses, replacing human evaluation entirely by assessment by machine is met with considerable cynicism [115]. A study published by Wang have shown that a combined approach can decrease human error by 85% for detection of breast cancer metastases in sentinel nodes [125]. Another important question that needs to be addressed is whether there is an actual decrease in overall turn-around time. Decreased ability to directly control diagnostic workflow and lack of clarity on amount of responsibility assigned to pathologists while reporting using AI are some practical issues that need to be resolved before a meaningful human-machine cooperation can occur in the clinical setting [91].

#### Future directions and opportunities

In the past few years, there has been an increase in the development of AI tools for detection of cancer by various companies like Visiopharm, Halo, Proscia, DeepLens, Inspirata and PAIGE.AI. Of these, Inspirata and PAIGE.AI are actively involved in creating large WSI repositories for training DL-algorithms [91, 126, 127]. FDA approval of the Philips whole-slide scanner in 2017 marked a watershed moment in the path towards digitization of clinical workflow [19]. The challenges thrown by the COVID-19 pandemic necessitated the adoption of digital workflow in daily clinical practice by some institutions, including ours at The Ohio State University Wexner Medical Center. Despite the myriad challenges and obstacles in the adoption of digital workflow replete with AI tools, there has been a paradigm shift in the landscape of digital pathology [91]. The advent of open-top light sheet microscopy which generates non-destructive, slide-less three-dimensional (3D) images of tissues might provide a substantially greater degree of spatial and architectural information needed for application of AI approaches. Similarly, MUSE microscopy might circumvent the need for tissue processing and staining by providing high-resolution images of tissues using ultraviolet rays [128]. While current AI applications can recognize tumor scores and grades, in the future, most of them will likely continue to be in the narrow AI domain, focusing on only a single task [129].

## Conclusions

The last few years have seen a tremendous growth in the development of novel AI approaches in pathology. These tools, when used intelligently, can improve diagnostic workflows, eliminate human errors, increase inter-observer reproducibility, and make prognostic predictions. While there has been an increase in the development of AI tools, the integration into clinical practice has somewhat lagged owing to several issues related to interpretability, validation, regulation, generalizability, and cost. As the need for personalized cancer care increases, AI applications may be implemented and used appropriately in conjunction with human pathologists, after standardized usage recommendations, and harmonization with current information systems. A multimodal approach using proteomics, genomics, and AI-based multiplexed biomarker quantifications, might be necessary for comprehensive patient-specific tumor precision therapy [91, 130].

## Data Availability

Not applicable.
